# Supplementary material for: Inflammatory factors and risk of lung adenocarcinoma: a Mendelian randomization study mediated by blood metabolites
Source: Front Endocrinol (Lausanne). 2024 Aug 27;15:1446863. doi: 10.3389/fendo.2024.1446863 (PMC11384989; doi:10.3389/fendo.2024.1446863)
Supplement: Supplementary file 1 [file DataSheet1.zip › supplementary files/S2/GCST90200391/forest.pdf]

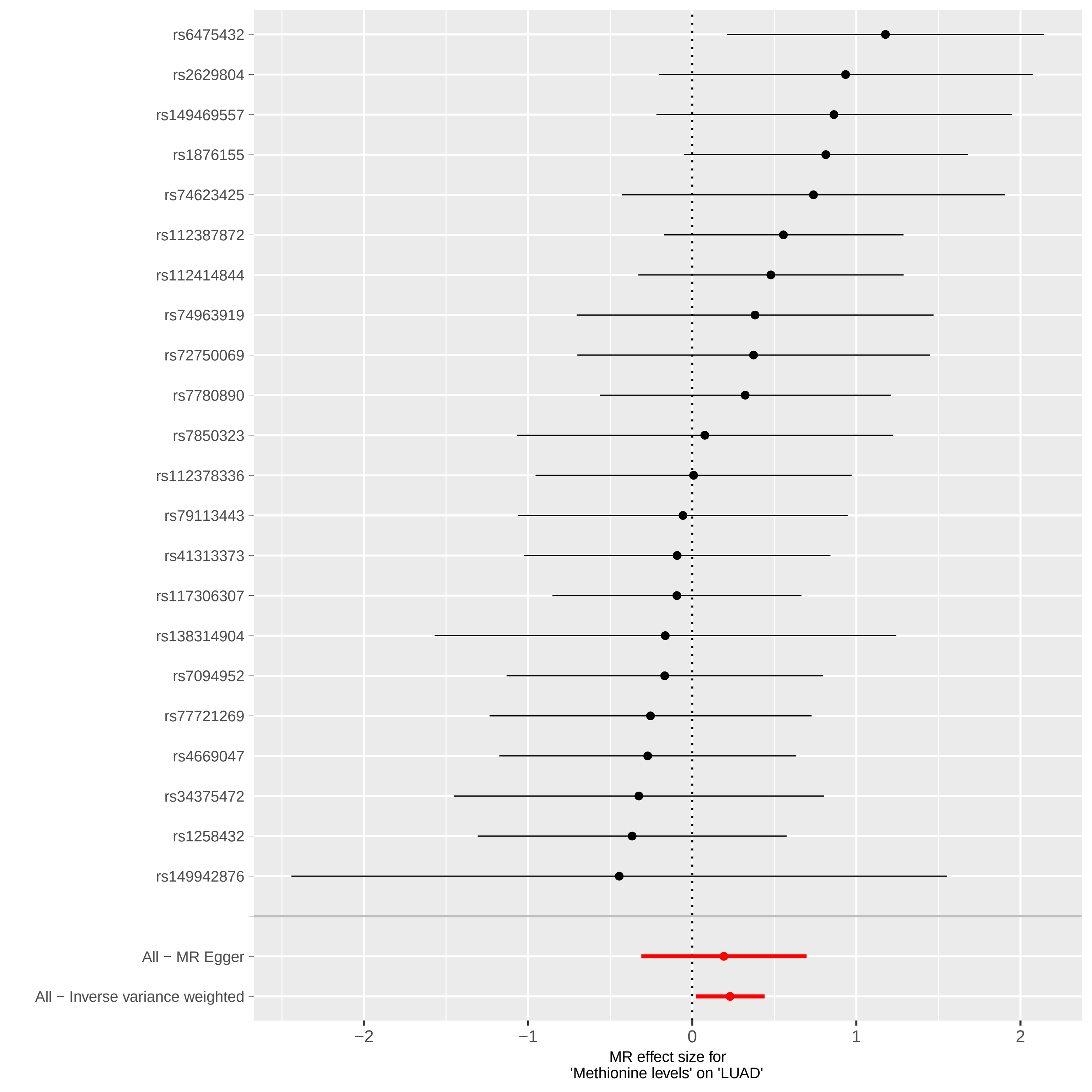

rs6475432
rs2629804
rs149469557
rs1876155
rs74623425
rs112387872
rs112414844
rs74963919
rs72750069
rs7780890
rs7850323
rs112378336
rs79113443
rs41313373
rs117306307
rs138314904
rs7094952
rs77721269
rs4669047
rs34375472
rs1258432
rs149942876
All − MR Egger
All − Inverse variance weighted
−2
−1
0
1
2
MR effect size for
'Methionine levels' on 'LUAD'
